# Expression of the gene encoding secretor type galactoside 2 α fucosyltransferase (FUT2) and ABH antigens in patients with oral lesions

**DOI:** 10.4317/medoral.17239

**Published:** 2011-12-06

**Authors:** Carlos Campi, Livia Escovich, Alejandra Moreno, Liliana Racca, Amelia Racca, Carlos Cotorruelo, Claudia Biondi

**Affiliations:** Laboratory of Immunohematology, Histocompatibility and Immunogenetics, Department of Clinical Biochemistry, Faculty of Biochemical and Pharmaceutical Sciences, National University of Rosario, Argentina. Suipacha 531. 2000 Rosario. Argentina. Department of Stomatology, Dentistry School, National University of Rosario, Argentina. Córdoba 3100. 2000 Rosario. Argentina

## Abstract

Objective: The aim of this work was to evaluate the expression of FUT2 gene in saliva and histo ABH antigens of patients with oral lesions. Study Design: In total 178 subjects were examined, half of whom suffered from oral pre-cancerous and cancerous lesions, while the other half were the healthy control group We analyzed the FUT 2 polymorphism by ASO-PCR (allele specific oligonucleotid – polymerase chain reaction) with specific primers for G428 allele and the wild type allele of FUT2 gene. To reveal A, B and H antigens in tissue sections of the patients (n= 89) we used a modified specific red cell adherence technique. Results: We found a high intensity of oral disease in the non-secretor group (OR = 2.43). A total of 58% of the patients with oral pre-cancerous and cancerous lesions was non secretors (se_/_), in contrast with the healthy population (21.5%). A strongly positive reaction was defined as a sheet of indicator erythrocytes adhered to the epithelial cells. In 31 of the 54 samples analyzed the test showed slightly positive results on atypical areas, and there was a complete antigen deletion in areas affected by neoplasia. Nineteen samples showed a total absence of ABH antigens in both histologically normal and pathological areas. Blood group antigens were expressed at a high level in benign and highly differentiated malignant tumors. In poorly differentiated malignant tumors, they were mostly absent. Conclusion: Considering these results we suggest the use of this method to monitor probable preneoplastic lesions in risk population, especially in those with no secretor status (absence of FUT2 gene).

** Key words:** FUT2 gene, ABH antigens, secretor status, oral lesions.

## Introduction

 Blood group antigens are polymorphic, inherited structures located on the red blood cell surface (1,2). The ABO blood group antigens are among the well-known fucosylated glycans. The expression of them is regulated by several glycosyltransferases that add monosaccharides to a precursor molecule in a sequential fashion (3-5). The α(l,2) fucosyltransferase that forms the H antigen, an essential precursor of the A and B antigens, plays a regulatory role in the tissue expression of the ABO antigens. 

The expression and secretion of ABO antigens in epithelial cells are controlled by secretortype α(l,2)fucosyltransferase activity, known as the Secretor (Se) transferase (FUT2 gene product) (2,6). Several different polymorphisms are known in the FUT2 gene, some called as silent mutations, while others as to non-functional enzymes. Homozygous individuals with non-functional enzymes are termed non-secretors (se_/_). About 20% of individuals are non secretors who fail to express the ABO antigen in saliva. On the other hand, heterozygous individuals carrying one functional allele, have secretion similar to the wild-type. These are termed secretors (Se) (6,7).

The functional significance for ABO antigen expression on erythrocytes has not been defined, but ABO-related structures may play a role in other systems. Alterations in the expression of fucosylated oligosaccharides have also been observed in several pathological processes, including cancer and atherosclerosis (8-10).

In carcinomas, altered expression of the various carbohydrate epitopes of this family occur, and are often strongly associated with either a good or bad prognosis. In human tumors, blood group antigens change in the same general direction as other glycosphingolipids do in tumors (11,12). The mechanisms of aberrant expression of blood-group antigens are not clear in all cases (13-15). The basis of the changes occurring in cell surface carbohydrate antigens during cellular differentiation and the further changes which occur with malignant transformation appears to be highly complex. It has been demonstrated in a number of earlier studies on the etiology and pathogenesis of certain diseases that the patients' secretor status (ABO (H) blood group antigens) may probably be a factor influencing the development of systemic oral diseases (16,17).

The aim of the present study was to evaluate the expression of FUT2 gene in saliva and histo ABH antigens of patients with oral lesions in order to determine whether this factor could be a marker risk of oral cancer.

## Material and Methods

 The patients analyzed in this study presented to the Stomatology Department of the Odontology Faculty of the National University of Rosario. Recruitment was made by consecutive sampling for a period of 18 months.

From a total of 178 subjects examined, half suffered from oral lesions (benign, pre-cancerous and cancerous ones), while the other half were the healthy control group. All were subjected to clinical oral examinations and standard evaluation tests in order to establish the secretor status of their saliva. In the group of patients with oral benign, pre-cancerous and cancerous lesions (experimental group), a histopathological examination of the oral mucosa was performed.

Patients with benign oral lesions showed hyperplasia caused by diverse agents such as infectious, inflammatory, traumatic, hormonal, and drugs. The premalignant lesions included leukoplakia and lichen planus. The malignant lesions studied were squamous cell carcinoma. 

All subjects gave informed consent to participate in the study, and the protocol was approved by the Ethic Committee of the School of Medical Sciences of Rosario, Argentina, according to the principles of the Declaration of Helsinki. 

 Serological studies

• Secretor status: we analyzed their saliva and serum by the agglutination inhibition technique (18).

• Specific red cell adherence test (19)

Slides of 4-5 micron section were deparaffinized and brought to water, immersed in tris buffered saline 0.05 M (pH 7.4) for 30 minutes, covered with isologous antisera according to patients’ blood group and incubated for one hour for A, B and O group in a moist chamber at room temperature. The slides were then dipped in tris buffered saline for three changes with occasional strings to remove the unreacted antisera. Few drops of 2-5% isologous indicator RBC’s suspension were added to the sections and incubated for 20 minutes in group A or B and one hour for group O. The slides were inverted over a support in a petridish containing tris buffered saline such that the undersurface of the slide just touched the solution and kept for five minutes to settle down unreacted RBCs. The slides were observed under the microscope with low power and photographed immediately.

Controls: Normal tissues containing blood group antigens, endothelium of blood vessels and RBCs acted as inbuilt positive controls and adipose tissues acted as inbuilt negative controls.

Interpretation: In the present study the isoantigenicity of epithelium was graded according to degree of adherence of indicator RBCs as strongly positive adherence (++++) to negative adherence (-). Levels intermediate were determined as 25% of adherence +, 50% of adherence ++ and 75% of adherence +++.

 Molecular studies 

 DNA isolation

Genomic DNA was isolated from saliva samples. We designed a protocol for DNA extraction from these samples. They were subjected to thermal shock by successive freezing and thawing and centrifuged to work with the cell button. We used the technique CTAB-DTAB (dodecyltrimethylammoniumbromide/ cetyltrimethylammoniumbromide adding CTAB directly without the addition of TE buffer (20,21).

The DNA concentration was measured spectrophotometrically at 260 nm and diluted in sterile water to a concentration of 100 ng per µL. 

 G428A polymorphism 

 The DNA samples were analyzed by ASO-PCR (allele specific oligonucleotid – polymerase chain reaction) with specific primers (Operon Lab) for G428 allele and the wild type allele of FUT2 gene. A fragment of 132 bp was amplified as described by Henry et al. (21), except for the annealing temperature modifications. According to gradient of PCR the Tm of the primers chosen was 66ºC. The PCR products (132 bp) were analyzed in 2 % agarose gel containing ethidium bromide. 

 Statistical Analysis 

 The categorical data were examined with a 2 test, and the ORs were calculated as measure of association. 

## Results

 In our population the nonsense mutation (428 G-A) in the FUT2 gene is the most frequent polymorphism. We studied the possible association between the 428 G-A in the FUT2 gene and oral disease progression. The genotyping revealed that 18 (20.5%) of the 89 blood donors were found to be non-secretors (se_/_) and 79.5 % of the healthy individuals studied presented the Se gene (FUT 2) that governs the secretion of water-soluble ABH antigens into saliva (control group). These secreted antigens can be demonstrated in saliva by agglutination inhibition tests with ABH antisera and molecular biology through analysis of the FUT 2 gene. In contrast, twenty-eight patients (58%) with oral pre-cancerous and cancerous lesions were non secretors, OR = 2.43; CI 95% (1.03; 5.71) (p= 0.0407) ( Table 1). We found a higher intensity of oral disease in the non-secretor group, and epithelial dysplasia was found exclusively in this group. 

**Figure 1:**
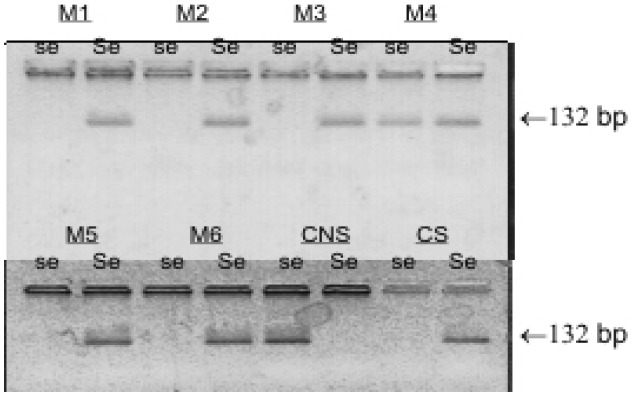
The agarose gel shows the PCR products of 132 bp for the M1-M6 samples. Each sample was analysed for the wild type allele (Se) and for G428A allele (se) and was run together with secretor control (CS) and non-secretor control (CNS).

The molecular analysis showed that 48.31% of patients was homozygous for the G428A mutation (the mutation present in the 2 alleles), while the other patients were homozygous for the secretor status (none of them presented the allele G428A), or heterozygous secretor (1 allele presented with the mutation G428A) (Fig. 1).

**Figure 2:**
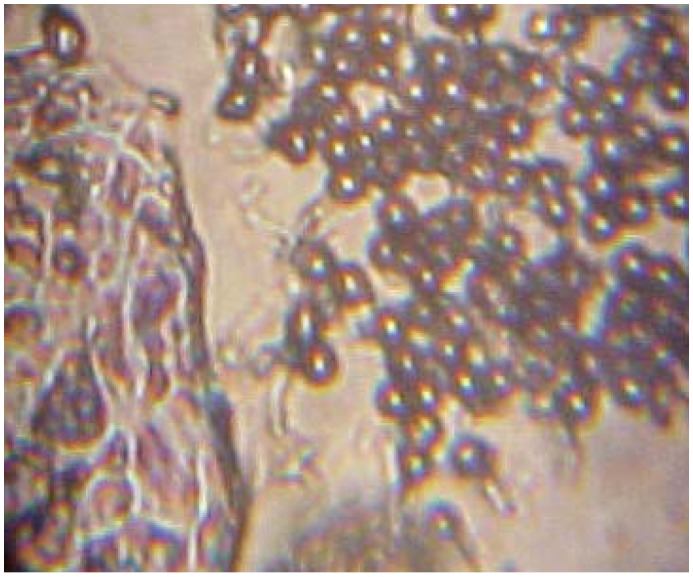
Cancerous lesion: non inmunoadherence of red blood cells.

**Figure 3:**
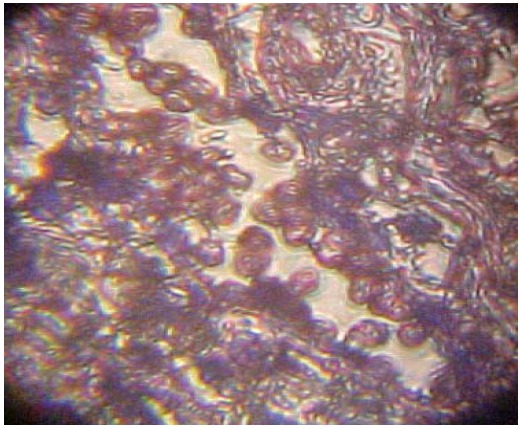
Oral benign lesion: inmunoadherence of red blood cells to the tissue.

Loss of A, B, and H antigens from the surface of tissue was observed in 31 patients with oral malignancies (57,41 %) ( Table 2). The other 42,59 % maintained the ABH expression ( Table 2). Thirty-nine percent of the histopathologically diagnosed benign lesions lost the antigenic reactivity (Fig. 2, 3).

**Table 1:**
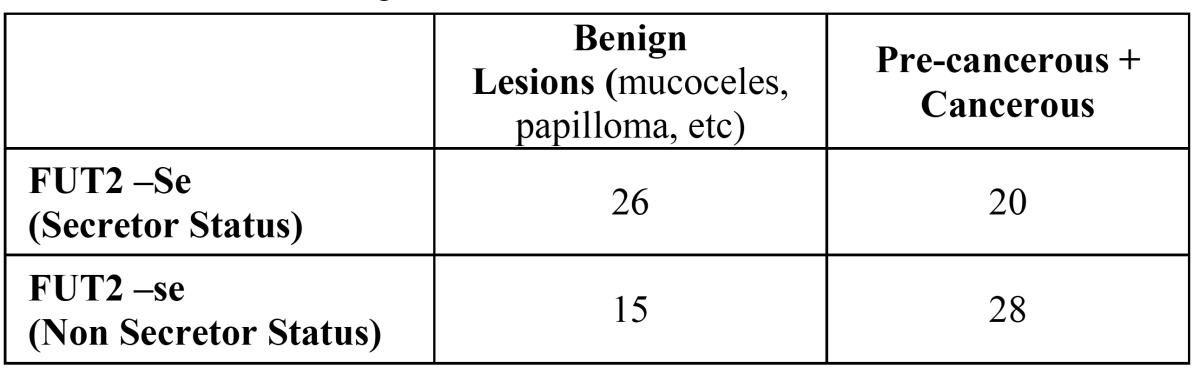
Secretor status in patients with oral lesions.

**Table 2:**
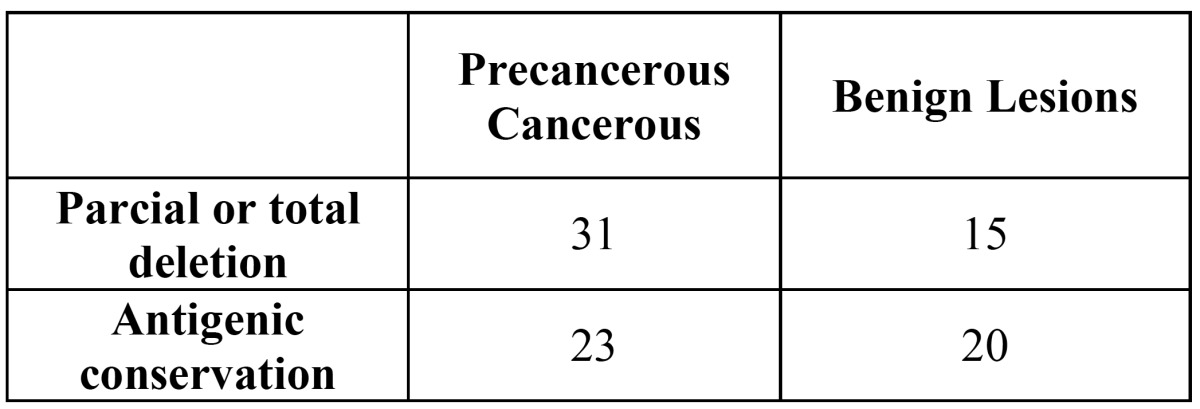
Abh antigens expression in fixed tissue sections of oral lesions.

Within the most invasive tumors sites, a deletion of ABH reactivity correlated significantly with the stage of tumor development and histological malignancy grade. 

In the sections studied, the endothelium of blood vessels was reactive with the erythrocytes (positive control) and adipose tissues did not react with the red blood cells (negative controls).

## Discussion

 The secretor status is defined by the presence of H type 1 antigen in body secretions such as milk and saliva. H type 1 antigen belongs to both the Lewis and the ABO (H) histo-blood-group systems and it is expressed in erythrocyte membranes and in several epithelial tissues. The secretor enzyme (FUT2), an α-1,2-fucosyltransferase, is responsible for the fucose transfer in an α-1,2 linkage to form the terminal H type 1 structure (3–6). The cell-surface fucosylated oligosacharides participate in several biological processes, such as embryogenesis, tissue differentiation, tumour metastasis, inflammation and bacterial adhesion (15,17). About 20% of the Caucasian population is non-secretor (9). Several disease correlations have been linked to non-secretor status. In general, being non-secretor results in several disadvantages regarding metabolism and immune function (4,8,14). 

Our results have demonstrated that most of the individuals examined in the healthy group were secretor (have the FUT2 gene) (79.5 %) and there were significant difference between secretors and non-secretors in the experimental group. We have also found a higher intensity of oral disease in the non-secretor group, and the occurrence of epithelial dysplasia was mostly found in the non-secretor group. This study evaluated the association between oral lesions and polymorphisms of the Se genes. We found that oral pre-cancerous and cancerous lesions were increased among individuals with non secretor status and nonsense mutation 428G→A (Trp143→stop) (58.33%). We found 20 patients diagnosed histopathologically as malignant lesions despite the secretory status.

The studies of patients with premalignant and malignant oral lesions, in which non-secretor status predominates, appear to be an associated risk marker for the development of oral cancer. Leukoplakia and erythroplakia are clinical changes in the oral mucosa regarded as potentially malignant lesions (1,9). Certain histopathological changes may indicate a malignant potential in a lesion. However, the presence of such changes is not a reliable predictor of malignant transformation, and their absence does not mean that the patient is out of risk of developing a tumour (1,13).

Carbohydrate structures were found on the cell surface bound to either lipid or protein embedded in the membrane. Changes in the carbohydrate structure of these cell-surface glycolipids and glycoproteins have been demonstrated during cell maturation development in adult tissue, and in relationship with malignant development (9-12). Blood-group antigens can be present on key receptors controlling cell proliferation, adhesion, and motility, such as epidermal growth factor receptor, integrins, cadherins, and CD-44. The patterns of expression of these various receptors differ according to the type of normal epithelium and the type of cancer, and therefore the role of ABH antigens in the biology of human cancers may also vary. The function of the ABO expression in normal stratified oral epithelium is unclear (16). Furthermore, the expression of histoblood-group antigens in normal human tissues depends on the type of differentiation of the epithelium and the maturation degree of the single cell within the epithelium. 

Although the relationship between epithelial dysplasia in a leukoplakia and malignant transformation of the lesion is debatable, many workers consider that the finding of epithelial dysplasia indicates a higher likelihood to develop malignancy. It is, however, more probable that the antigen changes found in the dysplastic lesions are associated with other factors, such as cell movement and growth rate, rather than malignancy per se (10). 

It is generally accepted that tumors are composed of heterogeneous cell populations with different biological behaviors. To obtain optimal prognostic information about the tumor, therefore, the entire tumor cell population should be studied. Despite the somewhat unrepresentative biopsy material, it was possible to show that loss of ABH antigens were associated with the tumor spread (stage). This could be of diagnostic and prognostic value. Similar loss of ABH antigens in bladder cancers was associated with a poor prognosis in some studies (22,23).

We also observed a reduction or complete deletion of A, B or H antigen expression in tissues sections of non secretor patients with oral pre-cancerous lesions or carcinomas. Disappearance of the antigens is ascribed to the absence of A or B transferase gene expression (24). These findings are consistent with several studies showing that the loss of A and B antigen expression is associated with increased tumourigenecity in syngenic animals. 

Other study has showed that the sequential expression of antigen is lost in carcinomas but retained in lesions with epithelial dys-plasia and in lesions which clinically and histologically are regarded as benign. It also showed that although the sequential expression of carbohydrate antigens are retained in lesions with epithelial dysplasia, these lesions differ from normal and benign lesions due to an extended distribution of one of the carbohydrate structures (12). Some findings have also demonstrated that malignant development in stratified oral epithelium is associated with aberrant glycosylation of cellular glycoconjugates and that there are differences between premalignant lesions and carcinomas which may prove to be of diagnostic significance (12).

To conclude, we propose that areas of SRCA-test negative epithelium are closely related to invasive carcinomas and may be their precursor lesions. In addition, our results indicate that at the same time as the morphological changes occur during the process of oral carcinogenesis, another series of events occurs. Further follow-up studies are required to clarify the role of predictive markers of risk in precursor lesions of oral cancer.
